# Design of Gelatin-Capped Plasmonic-Diatomite Nanoparticles with Enhanced Galunisertib Loading Capacity for Drug Delivery Applications

**DOI:** 10.3390/ijms221910755

**Published:** 2021-10-05

**Authors:** Chiara Tramontano, Bruno Miranda, Giovanna Chianese, Luca De Stefano, Carlo Forestiere, Marinella Pirozzi, Ilaria Rea

**Affiliations:** 1 Institute of Applied Sciences and Intelligent Systems—Unit of Naples, National Research Council, Via Pietro Castellino 111, 80131 Naples, Italy; chiara.tramontano@na.isasi.cnr.it (C.T.); bruno.miranda@na.isasi.cnr.it (B.M.); giovanna.chianese@na.isasi.cnr.it (G.C.); ilaria.rea@na.isasi.cnr.it (I.R.); 2 Department of Pharmacy, Università Degli Studi di Napoli Federico II, Via Domenico Montesano 49, 80131 Naples, Italy; 3 Department of Electrical Engineering and Information Technology, Università Degli Studi di Napoli Federico II, Via Claudio 21, 80125 Naples, Italy; carlo.forestiere@unina.it; 4 IEOS (Istituto per l’Endocrinologia e l’Oncologia Sperimentale) “G. Salvatore” Seconda Unità—CNR, Via Pietro Castellino 111, 80131 Naples, Italy; m.pirozzi@ieos.cnr.it

**Keywords:** diatomite, gold nanoparticles, gelatin, drug delivery, plasmonics, Galunisertib

## Abstract

Inorganic diatomite nanoparticles (DNPs) have gained increasing interest as drug delivery systems due to their porous structure, long half-life, thermal and chemical stability. Gold nanoparticles (AuNPs) provide DNPs with intriguing optical features that can be engineered and optimized for sensing and drug delivery applications. In this work, we combine DNPs with gelatin stabilized AuNPs for the development of an optical platform for Galunisertib delivery. To improve the DNP loading capacity, the hybrid platform is capped with gelatin shells of increasing thicknesses. Here, for the first time, full optical modeling of the hybrid system is proposed to monitor both the gelatin generation, degradation, and consequent Galunisertib release by simple spectroscopic measurements. Indeed, the shell thickness is optically estimated as a function of the polymer concentration by exploiting the localized surface plasmon resonance shifts of AuNPs. We simultaneously prove the enhancement of the drug loading capacity of DNPs and that the theoretical modeling represents an efficient predictive tool to design polymer-coated nanocarriers.

## 1. Introduction

Colorectal cancer (CRC) is one of the most tough-to-detect malignancies and the currently existing treatments are often inefficient to improve the patient’s quality of life [1,2]. The positive correlation between the expression of the transforming growth factor-β receptor I (TGF-β RI) and the progression of CRC is mediated by the TGF-β RI signaling pathway and results in uncontrolled cell proliferation and metastasis [3]. Several pharmacological approaches can block the TGF-β RI pathway and inhibit the metastatic progression of CRC, such as Galunisertib (LY2157299) [4]. However, the high-dose administration and fast metabolization of Galunisertib can be highly toxic due to the rapid generation of undesired metabolites in the plasma [5]. For this therapeutic agent, nanoscale delivery systems can protect the drug from the liver’s primary mechanism of metabolization, improving their therapeutic effect and reducing the administered dose [6]. Thanks to the progression of nanotechnology, a variety of nanocarriers have been proposed for disease therapy and diagnosis, including organic, inorganic, and liposome-based nanosystems [7,8].

Among inorganic silica nanoparticles (NPs), diatomite NPs (DNPs) can be obtained from the diatomite powder and display intriguing properties for therapeutic applications, such as the high surface-to-volume *ratio*, long half-life, and thermal and chemical stability [9]. Silica from diatom frustules was recognized by the Food and Drug Administration (FDA) agency as safe for human consumption (GRAS, 21 CFR 182.90), and classified by the Agency for Research on Cancer (IARC) in the 3rd group of “*Not classifiable as to its carcinogenicity to humans*”. The mesoporous structure and surface tunability of DNPs allow the development of drug delivery systems for precision medicine [9,10]. Moreover, when DNPs are decorated by metal nanostructures, such as gold nanoparticles (AuNPs), silica acquires the intriguing optical properties of AuNPs that could be used for combining drug delivery with biosensing [11,12,13], bioimaging [14], phototherapy [15], and other applications [16]. Indeed, when the size of a noble metal (i.e., gold) is confined at the nanoscale, a phenomenon known as Localized Surface Plasmon Resonance (LSPR) [17,18], arises. The coherent oscillation of electrons on the AuNP surface is responsible for the strong field enhancement in their surroundings and confers to hybrid systems composed of DNPs and AuNPs high sensitivity to local refractive index variations [12,19,20].

Recently, our group showed that when Galunisertib is encapsulated in DNPs capped by gelatin, the drug effect on CRC cells is strongly enhanced, allowing to lower the needed administered dose. Galunisertib was loaded in gelatin-capped DNPs with a drug loading capacity of 2 μg∙100 μg^−1^ of DNPs and an encapsulation efficiency (EE) of 0.4% [21]. Although in that study gelatin provided the nanocarrier with a pH-responsive and sustained drug release profile, we did not consider adjusting the desired drug loading capacity to the amount of gelatin in the shell surrounding DNPs. For this reason, to release the desired dose of Galunisertib in the cell, we increased the administered mass of DNPs within the limit of toxicity to cells. The drug loading capacity is a key factor in designing drug delivery systems [22,23] and it is worth also considering the possibility to improve this parameter without increasing the mass of NPs and toxicity. Therefore, since gelatin is a well-known biopolymer used in medicine [24,25], it is safer to modulate the amount of gelatin in the external shell rather than the mass of DNPs to improve the system drug loading capacity.

In this study, we fabricate DNP loaded with Galunisertib and capped by growing shells of gelatin to modulate the biosilica loading capacity according to the amount of gelatin in the external shell. To monitor the growth and degradation of gelatin shells spectroscopically, we decorate the surface of DNPs with gelatin-stabilized AuNPs (Gel-AuNPs). Here, gelatin is used in the synthesis of AuNPs to inhibit the formation of clusters and enable the construction of theoretical modeling based on a single NP. Moreover, in a subsequent step, gelatin is also used to induce the formation of an external shell around DNPs, which confer to our system the capability of retaining drug molecules and avoid drug dispersion phenomena [21]. To estimate the shell thickness of the AuDNP-LY@Gel system without costly instrumentations, we propose the reverse engineering of the gelatin thickness starting from the prediction of the LSPR response of Gel-AuNPs decorating the biosilica surface. Then, we theoretically estimate the plasmonic redshifts due to the average shell thickness and find a correlation between the gelatin experimental concentration and theoretical thickness. We demonstrate that the DNP loading capacity can be tuned by varying the amount of gelatin rather than the mass of DNPs and that both the formation and degradation of the shells can be monitored spectroscopically, overcoming the need for high-resolution instrumentation. Indeed, through the developed optical modeling we can extract information on different design parameters of optical drug delivery systems, such as the loading capacity, coating thickness, and drug release by monitoring the plasmonic resonance of the system.

## 2. Results and Discussion

### 2.1. Fabrication and Characterization of AuDNP-LY@Gel with Increasing Gelatin Shells

**Figure 1:**
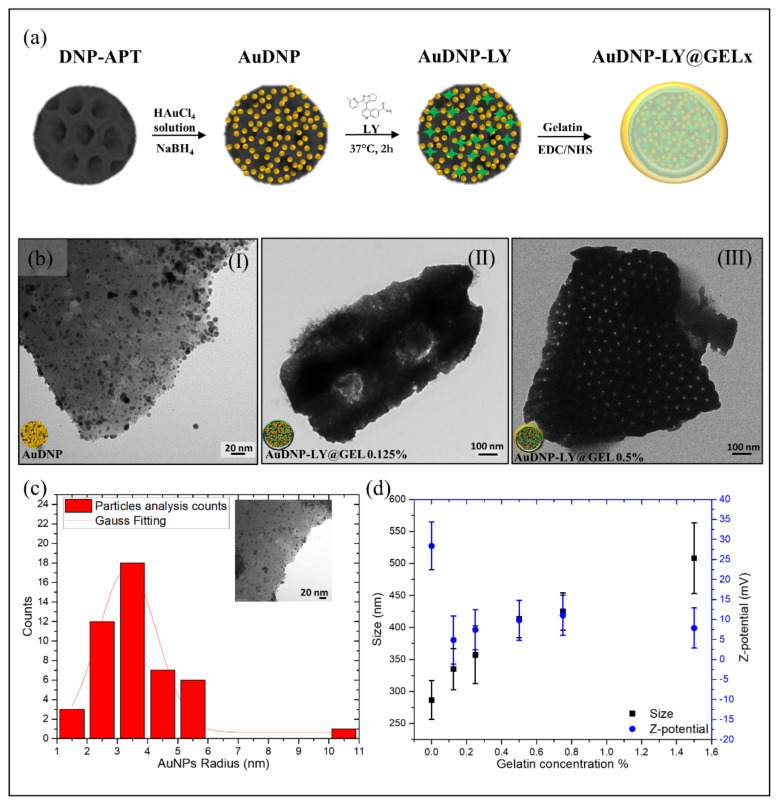
Preparation and Characterization of the AuDNP-LY@Gel system. (**a**) Schematic representation of the functionalization procedure. The growth of AuNPs on the surface of the DNP is achieved by dispersing amino-modified DNPs in the HAuCl_4_ solution and adding gelatin as a stabilizing agent. NaBH_4_ is used as the reducing agent. Then, the small molecule Galunisertib is loaded into the system and the gelatin shell is performed by crosslinking of the polymer matrix. The scheme is not in scale and not intended to represent the full sample composition. (**b**) TEM investigations of the AuDNP (I), AuDNP-LY@Gel_0.125%_ (II) and AuDNP-LY@Gel_0.5%_ samples (**c**). Particle size analysis of AuNPs decorating the surface of the DNP fitted by a Gaussian curve. (**d**) Change in the size (black) and surface charge (blue) of the samples AuDNP-LY@Gel according to the different gelatin amounts in the outer shell. The vertical bars are representative of the standard deviation (SD) on a minimum of three independent measurements.

The development of a hybrid nanosystem with increasing shells of cross-linked gelatin was performed according to the functionalization procedure reported in Figure 1a.

The growth of AuNPs on the surface of DNP (AuDNP) was performed through a liquid phase approach in which DNPs were suspended in a tetrachloroauric acid solution (HAuCl_4_) and the reduction of AuNPs occurred in-situ on the surface of DNPs. Before the growth of AuNPs, the surface of DNPs was modified with a 10% *v/v* APTES solution to anchor positive groups NH_3_^+^ on the biosilica surface (DNP-APT) and promote the electrostatic interactions between gold precursor ions and DNPs. The electrostatic attraction between negative gold precursors and the positive charged DNP (DNP-APT) granted an in-situ accumulation of AuNPs on the DNP-APT substrate. Briefly, DNP-APT were dispersed in 0.1 M HAuCl_4_ solution and stirred for ten minutes in presence of 0.025% gelatin as a stabilizing agent. The major advantage of gelatin as a stabilizer is that it can both tailor the nanocomposite properties and provide AuNPs with long-term stability preventing particle aggregation [26]. Furthermore, since gelatin is a natural biocompatible material its use as a stabilizing agent did not introduce any environmental toxicity or biological hazards in the final nanocarrier. Generally, gelatin-stabilized AuNPs are obtained by the reduction of gold salts in AuNPs under thermal heating until the dispersion color turns from yellow to red [27]. Here, for the first time, the reduction of gelatin-stabilized gold ions was performed by adding to the above dispersion a solution (0.1 M) of sodium borohydride (NaBH_4_). The size of the obtained AuNPs can be controlled by NaBH_4_ easier and faster than heating since the reducing agent is added dropwise. The formation of the DNP-AuNPs complex was immediately appreciated by a colorimetric change of the dispersion, which turned from light yellow (before reduction with NaBH_4_) to deep red (after reduction). The dispersion containing the hybrid system (AuDNPs) was washed intensively to remove excess reagents and resuspended in PBS solution. Since, in the complex AuDNP, the electrostatic attraction between gelatin-stabilized AuNPs and DNP-APT is not stable under harsh stirring and pH conditions, we operated a cross-linking of the gelatin stabilizing AuNPs to ensure their stability on the surface of DNP-APT. To this aim, the system AuDNP was dispersed in 1-ethyl-3-(3-dimethyl aminopropyl) carbodiimide (EDC)/N-hydroxysulfosuccinimide (NHS) solution to promote the peptide bond between amino (NH_2_) and carboxyl groups (COOH) on the gelatin backbone. The crosslinking of the gelatin stabilizing AuDNP dispersion strengthened the presence of AuNPs on the biosilica surface, allowing to further functionalize the complex and prevent loss of AuNPs on the substrate. At this stage, gelatin must be considered only as a surfactant that inhibits particle aggregation and improves the AuNP stability on biosilica. Then, Galunisertib (LY) was loaded in the hybrid complex through both entrapping and physisorption methods. To prevent the burst release effect attributed to porous drug carriers [28], the complex AuDNP-LY was mixed with high-concentrated solutions of gelatin to grow a shell around the whole system. To this purpose, the AuDNP-LY complex was dispersed in gelatin solutions (0.125%, 0.25%, 0.50%, 0.75%, 1.5% *p/v*) and crosslinked via EDC/NHS chemistry that favored the development of shells with different thicknesses. At the end of the functionalization procedure, the gelatin shells covered the entire hybrid system composed of DNP decorated by gelatin stabilized AuNPs.

To assess the presence of the outer gelatin shell in the AuDNP-LY@Gel complex, transmission electron microscopy (TEM), Dynamic Light Scattering (DLS), and ζ-Potential analysis were performed. According to Figure 1b(I), the irregular surface of DNPs was decorated by a carpet of AuNPs with a mean radius *r* of ~ 3 nm (Figure 1c). The presence of the gelatin shell covering the final AuDNP-LY@Gel_x_ complex was confirmed by the surface of the complex becoming darker and smoother upon an increment of the gelatin concentration [29,30]. As evident from Figure 1b(II,III), the gelatin shell on the biosilica surface covered the carpet of AuNPs, which were no longer visible by TEM analysis. Additionally, the porous structure of DNPs in Figure 1b(III) is more evident than in Figure 1b(II), due to the heterogeneity of the diatomite powder that is constituted by various species of diatoms having diverse pore-pattern (see also Figure S1). According to DLS and ζ-potential investigations (Figure 1d), DNPs-APT were characterized by a mean diameter of 290 (30) nm and a charge of 28 (6) mV due to the presence of protonated NH_3_^+^ groups on their surface. Increasing concentrations in the gelatin shell caused an increment in the particle size mainly ascribed to the steaky nature of gelatin and the formation of agglomerates between the DNPs. In the sample AuDNP-LY@Gel_0.125%_ and AuDNP-LY@Gel_0.25%,_ the size increment observed was almost similar to the control without the gelatin shell with a variation of about 30 nm. For the AuDNP-LY@Gel_0.5%_ sample, a significant variation of size from 290 (30) to 420 (60) nm was appreciated and even a greater variation was detected in the AuDNP-LY@Gel_1.5%_ sample. In fact, by increasing the amount of gelatin in the external shell, the internal cross-linking reactions occurring between the DNPs arise and cause particle agglomeration in the samples [31]. Therefore, in the sample with the highest amount of gelatin in the shell a size increment of about 250 nm was observed compared to the control sample. The size evolution observed after the formation of the gelatin shell was also justified by the surface charge of the samples, which was close to zero [31]. The type B gelatin used in this study has an Isoelectric Point (IEP) between 4.8 and 5.4, therefore it was expected for AuDNPs-LY@Gel samples to have a negative surface charge in an aqueous solution pH 6.5–7.0 (pH >IEP). However, a shift of the IEP of the gelatin toward 6–7 can be observed as a consequence of the crosslinking mechanism between COOH and NH_2_ groups in the polymer backbone [32]. Due to this slight shift, the surface charge of AuDNP-LY@Gel samples was expected to be neutral at the IEP, or slightly positive. The charge of AuDNP-LY@Gel complex (0.125%, 0.25%, 0.5%, 1%, and 1,5%) was comparable to each other’s and decreased from 28 (6) (DNPs-APT) mV to a mean value of 7 (5) mV in an aqueous solution, confirming the successful cross-linking of the gelatin shell. The small ζ-potential value caused in the samples particle aggregation and flocculation that lowered the repulsion forces between AuDNPs-LY@Gel_x_ complex and the physical colloidal stability as well. Since the Gel-AuNPs composing the system were firmly stabilized on the DNP surface, these aggregation phenomena did not affect the stability and plasmonic response of AuNPs.

### 2.2. Reverse Engineering-Based Modeling of the LSPR of AuDNPs-LY@Gel Drug Delivery System

**Figure 2:**
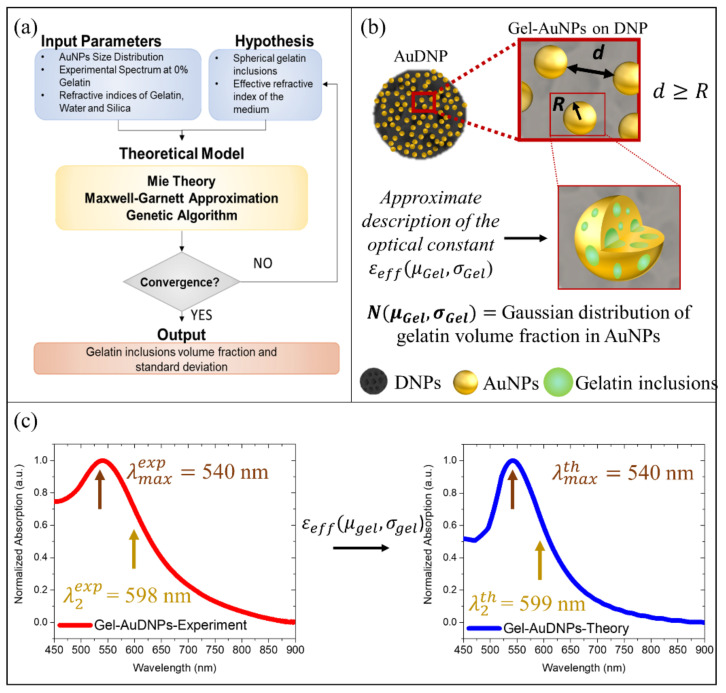
Reverse engineering problem: (**a**) Schematics of the combined theoretical and experimental approach followed to derive the volume fraction of the gelatin inclusions. (**b**) Schematics showing the approximations followed in the optical description of hybrid gel-AuNPs. (**c**) The normalized experimental absorption spectrum of Gel-AuDNPs with no gelatin shells (red line) having LSPR $\lambda_{2}=540\;\mathrm{nm}$ and inflexion point $\lambda_{2}=598\;\mathrm{nm}$; normalized theoretical absorption spectrum of gel-AuDNPs with no gelatin shells obtained by the application of the parameters $\mu_{gel}=0.11$ and $\sigma_{gel}=0.03$ (blue line). The minimized root mean square between theoretical and experimental spectra is 0.09.

The morphological characterization reported in the previous section provided the first evidence of the increase of cross-linked gelatin shells on AuDNPs-LY@Gel systems. Unfortunately, an accurate estimation of this parameter, which could be crucial in the design of DNPs-based drug delivery systems with enhanced drug loading capacity, could not be provided by TEM micrographs or DLS measurements since the dimensions of DNPs, on the one hand, gelatin-stabilized AuNPs (Gel-AuNPs) and shell thicknesses, on the other hand, were significantly different. For this reason, to extrapolate our missing information (i.e., gelatin shell thickness *t*), we exploited the LSPR optical response of AuNPs providing full modeling to our systems, starting from a reverse engineering approach. The small AuNPs, grown in-situ on the DNP surface and stabilized in presence of gelatin, exhibited a mean radius *r* of ~3 nm, as highlighted in Figure 1c. Moreover, TEM micrographs clearly show that, on average, their spacing was sufficiently larger than their size. For this reason, they can be considered as non-interacting NPs in good approximation (Figure 2b). However, the application of the simple Mie theory model to very small AuNPs dispersed in water would result in an LSPR peak ($\lambda_{max}$) located at a wavelength of ~515 nm in the visible spectrum. Instead, from the absorbance spectroscopy performed on the AuDNPs-LY systems with no further gelatin coatings, the typical plasmonic response of AuNPs was observed, but with a huge redshift (~25 nm) of the resonance peak ($\lambda_{max}\;$~540 nm) (Figure S2). We attributed this shift to the combination of two parameters. First, the reduction of AuCl_4_^−^ ions in presence of the gelatin, which led to the formation of inhomogeneous AuNPs with a significant percentage of gelatin remaining entrapped in the volume during nucleation and growth processes (Figure S1b). This resulted in a first resonance shift to 525 nm (Figure S2). Secondly, the presence of DNPs as substrates in the surroundings of the plasmonic NPs, which possess an effective refractive index higher than water, caused a further resonance shift to 540 nm. Although Gustav Mie found the analytical solution of Maxwell’s equations for the scattering and absorption cross-sections of a pure metallic nanosphere [33,34], his description did not consider the use of stabilizing agents and inhomogeneities occurring during the bottom-up synthesis of NPs. Stabilizers, as gelatin, having their refractive index, are generally involved in the chemical synthesis of colloidal NPs. Their use affects the effective dielectric constant and optical properties of the hybrid NPs, making an accurate description of their absorption spectra difficult. The effect of inhomogeneities, denoted here as inclusions, on the optical constants of the hybrid material can be predicted by Maxwell-Garnett’s homogenization theory [35]. Therefore, we combined Mie Theory and Maxwell-Garnett approximation to predict the absorption and scattering cross-sections of our Gel-AuNPs [36]. We introduced an effective dielectric constant $\varepsilon_{eff}(\mu_{Gel},\sigma_{Gel})$ describing the hybrid nature of Gel-AuNPs, in which the gelatin inclusions are assumed as spherical for sake of simplicity, and an effective refractive index of the medium, which considered the anchoring of the Gel-AuNPs on DNPs.

More precisely, we assumed the gelatin inclusions as a gaussian distribution of spherical particles within the AuNPs, as schematized in Figure 2b, and we optimized the mean $\mu_{Gel}$ and standard deviation $\sigma_{Gel}$ of the volume fraction occupied by the inclusions with a simple optimization strategy. We used the Genetic Algorithm (GA) to minimize the objective function reported in Equation (4), imposing a stopping condition below 0.1 and extracting the two fitting parameters, namely the mean volume fraction percentage occupied by gelatin inclusions (modeled as spheres) within Gel-AuNPs ($\mu_{Gel}$) and the standard deviation of these spheres ($\sigma_{Gel}$). The root mean square value between theoretical and experimental absorbance spectra of AuDNPs-LY returned by GA was 0.09 (<0.1) and the fitting values were 0.11 and 0.03 for $\mu_{Gel}$ and $\sigma_{Gel}$, respectively. This optimization gave us three important results: first, Gel-AuNPs contained a gelatin volume fraction of 11±3%, which was in good agreement with the initial weight *ratio* (*w/w*) between gelatin and HAuCl_4_ of the reaction volume; secondly, we obtained an estimation of the medium refractive index from which Gel-AuNPs optical response was affected, corresponding to a 10% silica (DNPs) and 90% water; finally, accurate optical modeling of the hybrid AuDNPs-LY system immersed in water was obtained, confirming the consistency of our initial hypotheses. The model is strongly based on the homogenization approach, which was possible due to the very small size of the AuNPs compared to the operating wavelength. The description of Gel-AuNPs would safely hold also in case of the presence of gelatin only on the surface of the AuNPs and not also within, thus offering an accurate description of the hybrid system (see also Figure S2).

As shown in Figure 2c, the theoretical modeling of the hybrid AuDNPs system is in very good agreement with both mean LSP resonance $\lambda_{max}^{exp}$ and the inflection point $\lambda_{2}^{exp}\;$of the experimental absorption spectra, corresponding to 540 nm and 598 nm, respectively. The inflection point $\lambda_{2}$ is simply the minimum of the first derivative of plasmonic absorbance spectra and will be crucial in the next section to increase the sensitivity of AuDNPs-LY@Gel systems. The variation of 9% between theory and experiments resulting from these simulations was attributed to the different absorption intensities between 450 and 500 nm, which arose experimentally from both LY and gelatin showing non-negligible absorptions in the UV and visible region (450–500 nm) of the spectrum. However, both $\lambda_{max}$ and $\lambda_{2}$ were not affected by absorptions in that region and, consequently, they could be considered negligible. The small molecule LY, having a refractive index of 1.75, was not considered in the model optimization since the low molecular weight and tiny concentration could not result in LSPR shifts. This hypothesis was confirmed by experimental absorption measurements, which are reported in Figure S3 of the Supplementary Materials.

### 2.3. Correlation of Gelatin Concentration with Mean Shell Thickness on AuDNPs-LY@Gel Drug Delivery System Based on a Validated Optical Model

**Figure 3:**
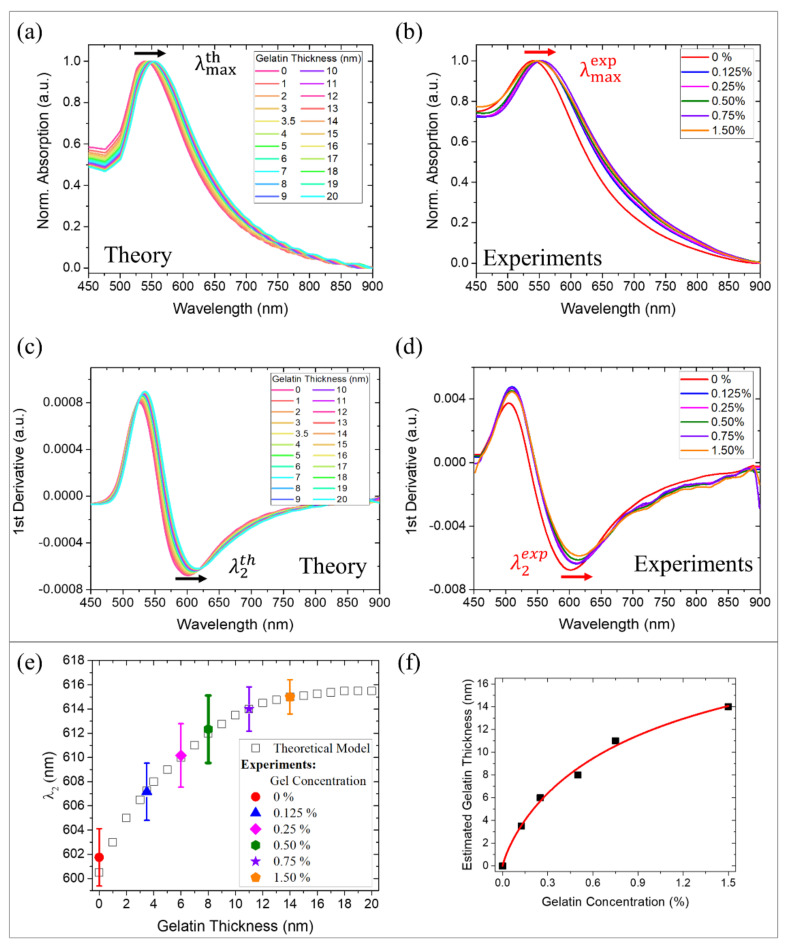
Correlation between gelatin concentration and thickness: (**a**–**c**) theoretical absorption spectra and first derivatives of AuDNPs with increasing gelatin shell thicknesses. The black arrows (redshifts of (**a**) $\lambda_{max}^{th}$ and (**c**) $\lambda_{2}^{th}$) are associated with the increase in the gelatin coating thickness. (**b**–**d**) Experimental absorption spectra and first derivatives of AuDNPs with increasing gelatin concentrations: 0% (red), 0.125% (blue), 0.25% (purple), 0.50% (green), 0.75% (violet), 1.50% (orange). The red arrows (redshifts of (**b**) $\lambda_{max}^{exp}$ and (**d**) $\lambda_{2}^{exp}$) are associated with increasing gelatin concentrations. (**e**) Comparison between $\lambda_{2}^{th}$ versus gelatin-coating thickness (theoretical model, white squares) and $\lambda_{2}^{exp}$ versus gelatin concentration (experimental data, colored squares). (**f**) Correlation between the gelatin concentration and estimated coating thickness. The red line is a fitting of the scatter plot (Equation (1)).

Once the goodness of our model was assessed in terms of accurate prediction of the optical response ($\lambda_{max}$ and $\lambda_{2}$) of Gel-AuNPs on DNPs in presence of LY, we applied it to the AuDNPs-LY@Gel_x_ systems to give an estimation of the gelatin shell thicknesses as a function of the increasing gelatin concentrations. The correlation between gel concentrations and effective gelatin shell’s average thicknesses (t) can be a crucial parameter for the design of a nanocarrier and, employing our optical model, we provided our system with an efficient strategy to monitor the shell formation. First, we simulated the LSPR response of the AuDNPs-LY systems with shells of increasing thicknesses (*t*) in the NPs surroundings. By doing this, we modified the effective refractive index of Gel-AuNPs on the DNPs surface observing, as expected, a plasmon redshift with the increasing gelatin shell thickness. Indeed, crosslinked gelatin possesses a refractive index higher than water and silica, thus causing a redshift of the plasmon resonance $\lambda_{max}$. We performed the simulation for average thicknesses of gelatin shells in the Gel-AuNPs surroundings in the interval (0–20) nm and the results are reported in Figure 3a. Differently, we experimentally monitored the plasmon resonance shift corresponding to the chemical crosslinking of different gelatin concentrations on the AuDNPs-LY@Gel_x_ systems (Figure 3b). Since we were dealing with very tiny AuNPs, we also considered the inflection point $\lambda_{2}$ of both theoretical and experimental absorption spectra, which enabled the enhancement of the plasmonic response sensitivity [37,38]. To do this, we calculated the first derivatives of the spectra and took, as $\lambda_{2}$, the minima of these curves (Figure 3c,d). Although there was another inflection point ($\lambda_{1}$) corresponding to the maxima of the first derivatives, it has been already demonstrated that the highest sensitivity for refractive index sensing can be obtained by considering the $\lambda_{2}$ values [37,38]. Moreover, as explained earlier, the first inflection points lie near the regions in which the theoretical prediction was not as accurate as in the other regions due to the neglected absorptions of LY and gelatin in those regions (Figure S4). For these reasons, from this point on, we only consider the inflection points for our discussion. From the theoretical point of view, $\lambda_{2}$ underwent a redshift as a function of the gelatin thickness. The $\lambda_{2}$ exhibited a red shift from 600 to 616 nm following a saturation curve with a linear range from 0 to ~11 nm of gelatin thickness and achieving saturation at ~18 nm (white squares in Figure 3e). This saturation behavior can be easily explained by the rapidly decaying field enhancement of Gel-AuNPs, whose plasmonic effect is localized to the NPs surroundings.

This means that, even if thicker gelatin shells could be easily simulated, the inflection points of the absorption spectra would have been not affected anymore by the increase of the gelatin shell. Therefore, we stopped our simulations at 20 nm gelatin thickness. Meanwhile, the experimental evaluation of $\lambda_{2}\;$was performed by measuring the absorption spectra of the AuDNP-LY@Gel systems obtained by crosslinking different gelatin concentrations: 0%, 0.125%, 0.25%, 0.5%, 0.75%, and 1.50% on the AuDNP-LY systems. Accordingly, a redshift of the inflection points was observed for the different gelatin concentrations. The theoretical and experimental results reported in Figure 3c–e allowed the introduction of a correlation between gelatin concentration and gelatin thickness on the hybrid AuDNPs-LY delivery system. This relationship between gelatin concentration $\left[c_{gel}\right]$ and gelatin thickness (t) exhibited a saturation behavior due to the electromagnetic field decay in the surroundings of AuNPs, which could be described by a Michaelis-Menten type equation in the investigated thickness range (Figure 3f):

(1) $$t=t_{max}\frac{\left[c_{gel}\right]}{k_{t}+\left[c_{gel}\right]}$$

where $t_{max}$ ~ 18 nm is the maximum gelatin thickness at which a redshift of the inflection point $\lambda_{2}$ of the absorption spectra of Gel-AuNPs was still detectable and $k_{t}=0.63\%$ is the gelatin concentration at which the shell thickness on the AuDNP-LY@Gel system corresponded to half of the $t_{max}$ (~ 9 nm). Therefore, we found a strong analogy between the formation of gelatin shells on the Gel-AuDNPs and the typical kinetics of substrate-enzyme reactions. In this analogy, the gelatin concentration acts as the substrate, while the electromagnetic field decay in the surroundings of AuNPs acts as the limiting factor of the process (enzyme). While saturation in a typical enzyme-substrate reaction is achieved when the enzyme is exhausted, in our case saturation is achieved when the electromagnetic field enhancement in the Gel-AuNPs surroundings is decayed. At fixed times, we correlated the shell thickness directly with the gelatin concentration according to the same equation. After a linear increase of the estimated thickness as a function of the gelatin concentration, we observed a saturation, which is not due to the gelatin formation itself but to the plasmonic response of Gel-AuNPs on DNP, which is not sensitive any more to the gelatin formation. The modeling and estimation of the average gelatin thickness of our drug delivery system was revealed as fundamental in the nanocarrier design. Indeed, the model provided the reverse design of a system in which the gelatin concentration could be tuned to achieve the desired average coating thickness.

### 2.4. Evaluation of the Loading Capacity of the Nanosystem AuDNP-LY@Gel_x_

**Figure 4:**
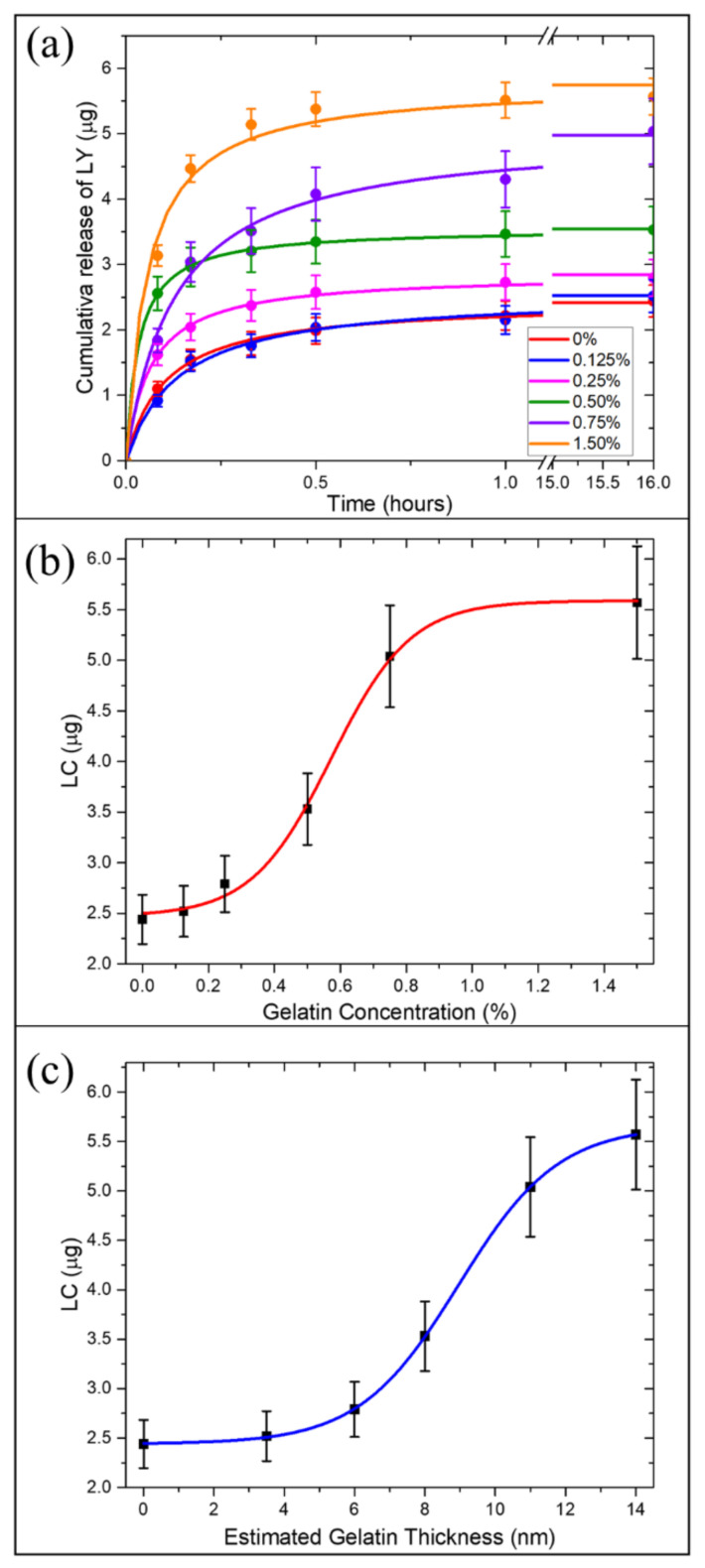
Correlation of the drug loading capacity of AuDNP-LY@Gel_x_ with the gelatin shell. (**a**) Cumulative release of LY from AuDNP-LY@Gel samples with increasing concentration of the gelatin shell in PBS solution containing trypsin enzymes (60 μg/mL). (**b**) Experimental estimation of the drug loading capacity in response to the concentration of the gelatin shell. (**c**) Theoretical estimation of the drug loading capacity according to the increasing thickness of the gelatin shell. The vertical bars are representative of the SD on a minimum of three independent measurements.

The burst release of a significant fraction of payload in the medium within a few minutes represents the most frequent drawback of porous drug carriers. Such uncontrolled release reduces the effective nanocarrier drug loading capacity and, in turn, the amount of drug delivered to the desired site of action [39]. To avoid this limitation, porous drug carriers can be encapsulated in polymeric matrices able to retain the therapeutic compound and control the drug release in response to external *stimuli.* We already assessed the ability of a gelatin shell embedding drug-loaded DNPs to allow a sustained release of the drug in response to the acidic microenvironment [21]. However, we did not investigate the loading capacity (LC) tunability of porous DNPs as a function of the amount of gelatin in the external shell. To this aim, in this study, in vitro-drug release analysis was performed in triplicate and the amount of drug released in the solution was quantified through Reversed-Phase High-Performance Liquid Chromatography (RP-HPLC) (Figure 4a). Drug release investigations were carried out in Phosphate Buffered Saline (PBS) solutions with a large excess of trypsin enzyme for 16 h. Recent findings suggested that gelatin is preferentially degraded by trypsin compared to enzymes such as lipase and amylase [40]. Moreover, since trypsin overexpression is involved in tumor progression and metastasis, in vitro release tests were carried out in presence of a large amount of trypsin to simulate the cancer microenvironment [41]. The LC and encapsulation efficiency (EE) of AuDNP-LY@Gel_x_ with increasing gelatin amount in the outer shells was compared to the AuDNP control sample (in which gelatin was used only as a stabilizer agent in the AuNP synthesis, but not as the external shell). We evaluated the influence of the shell thickness (estimated in previous sections) on the system loading capacity. First, we measured the LC and EE of the control AuDNP and found that, even without the external gelatin shell, both LC and EE (2.4 ±0.2 μg and 0.48% respectively) were higher than the previous LY delivery system [21]. This improvement can be ascribed to the presence of gelatin as AuNP stabilizer and its ability to establish strong interactions with drug molecules [42]. Therefore, it was already possible to appreciate an improvement of the DNP drug LC and EE by modifying the AuNP synthesis approach. Later, we studied the variation of the LC and EE as the concentration of gelatin increased, figuring out a way to modulate the desired LC to the shell grown on the DNP surface. According to Figure 4a, the increase of gelatin concentration/thickness in the shell solution resulted in a higher amount of LY entrapped in the system and available for delivery to the desired site of action. The AuDNP-LY@Gel_0.125%_ exhibited an EE of 0.50% (LC was 2.5 ± 0.3 μg) similar to the control, probably because the used gelatin concentration was too low to improve the LC of the nanosystem. Therefore, no differences in the amount of loaded and released drug were detected compared to the control sample. Conversely, when the gelatin concentration increased from 0.125% to 0.25%, the ability of the gelatin shell to retain LY increased, and the LC of the sample was improved up to 2.8 ± 0.3 μg of the drug, with an EE of 0.56%. An even higher LC and EE of LY were observed in the samples AuDNP-LY@Gel_0.5%_ and AuDNP-LY@Gel_0.75%_, respectively $3.5\;\pm \;0.4\;\left(EE=0.70\%\right)$ and 5.23 ± 0.5 μg (EE=1.04%), confirming that the higher was the concentration in the gelatin shell, the higher the amount of drug entrapped in the delivery system.

The LC swiftly approached the saturation point in the sample AuDNP-LY@Gel_1.5%_, which released about 5.6 ± 0.6 μg of LY ($LC_{max},\;EE=1.12\%$). All the samples displayed a similar release profile with 50% of the drug released within 20 min, due to the rapid degradation of the gelatin shell by the serine-protease enzyme trypsin. The excess of trypsin in the medium digested the gelatin shell rapidly and did not allow us to appreciate the release kinetics of LY from the gelatin-capped samples. However, our group already investigated the drug release profile of gelatin-capped drug carriers in previous investigations [21]. However, we demonstrated that the presence of gelatin capping the biosilica surface provided the system with pH-responsive properties (Figure S5), due to the enhanced extension of the gelatin chains in acidic microenvironments [43]. The therapeutic dose of a drug to encapsulate in carriers is generally a non-modifiable parameter, and it is not obvious that NPs have the adequate LC for encapsulating the therapeutic dose. In this scenario, to reach the wanted amount of drug released, the quantity of administered NPs must be raised. The opportunity to select the desired amount of drug by varying the gelatin shell is a great opportunity when DNPs are employed as carriers of chemotherapeutic molecules. Therefore, the tunability of LC as a function of the gelatin shell represents the means to improve the LC of DNPs without increasing the mass of administered biosilica.

The variation of the LC in response to the increasing concentration of gelatin in the outer shell followed a typical sigmoidal behavior (Figure 4b). The amount of LY retained in the system did not show significant variation for gelatin concentrations below 0.125% (toe region), since the gelatin shell was not thick enough to appreciate an improvement of the loading capacity compared to the control. As is obvious from Figure 4c, the drug loading capacity of AuDNP-LY@Gel_x_ systems followed the same sigmoidal curve as a function of the gelatin estimated average thickness. No significant enhanced retaining efficiency was observed for gelatin average thickness below ~6nm. At higher gelatin concentrations, instead, a direct proportionality with the nanosystem drug loading capacity was observed, reaching a plateau region in the sample with a gelatin concentration of 1.5%.

The increment of the LY retention in the system can be explained by considering the non-covalent interactions between the gelatin molecules and LY. The type-B gelatin shows an IEP value between 4.7–5.2 and displays an almost neutral charge at a pH value of 4.5. When the system was dispersed in the gelatin solution pH 4.0, the LY molecules physisorbed on the surface only exhibited a negligible positive surface charge on the quinoline-carboxamide group. Therefore, by increasing the gelatin concentration, the strength of Van der Walls interactions occurring between the drug molecules and polymer chains increased on the surface of the system, allowing better retention of the LY. Moreover, by raising the gelatin concentration, a larger polymer matrix was crosslinked as well, thus inhibiting the diffusion of the drug molecules out of the system. The sigmoidal relationship between the drug LC (μg) of AuDNPs-LY@Gel_x_ systems and the estimated gelatin shell thickness (t, nm) can be expressed as a Boltzmann equation:

(2) $$LC=LC_{max}+\frac{\left(LC_{min}-LC_{max}\right)}{1+e^{\frac{t-\left(\frac{t_{max}}{2}\right)}{\Delta t}}}$$

where $LC_{max}$ and $LC_{min}$ are the maximum and minimum amounts of LY loaded on the AuDNP-LY@Gel_x_ systems reported previously, *t* is the estimated gelatin thickness, $t_{max}\;\sim \;18\;\mathrm{nm}$, and Δt is:

(3) $$\Delta t=\frac{LC_{max}-LC_{min}}{4{\left.\frac{d\left(LC\right)}{dt}\right|}_{t=\frac{t_{max}}{2}}}$$

The LC trend of the AuDNP-LY@Gel_x_ systems as a function of the estimated gelatin thickness reported in Figure 4c exhibited three different regimes, which can be described with the Boltzmann type equation (Equation (2)): a toe region in which the gelatin thickness did not affect the LC capacity of the system (0–5 nm); a linear region in which the LC was directly proportional to the estimated gelatin thickness (5–11 nm) due to the increase of the interactions between the drug and gelatin molecules; and a saturation region (11–14 nm) in which the drug, whose experimental concentration was kept constant ($1\;\mathrm{mg}\;\cdot \;{\mathrm{mL}}^{-1}$), represents the limiting factor of the entrapment efficiency in the gelatin matrix. It is worth mentioning that the drug loading capacity enhancement due to the increased shell thickness could be fully considered in the estimation model we described earlier.

Surprisingly, the saturation achieved by the drug loading enhancement fell in the range of the estimated gelatin thickness (0–20 nm). As stated in the previous section, this was not obvious since our system could have reached saturation in drug loading capacity for gelatin thicknesses higher than 18 nm, in which no plasmon resonance shifts would have been observed. In that case, our optical description of the system would have been valid only for a limited part of the work. Instead, we provided our system with complete optical modeling, in which the estimated gelatin thickness could be experimentally verified and tuned in time to monitor two parameters: the gelatin shell formation (with an estimation of its thickness) via simple spectroscopic measurements, and the simultaneous gelatin degradation and drug release. This second part will be discussed in-depth in the next section.

### 2.5. Application of the Model to the Monitoring of the In-Situ LY Drug Release

**Figure 5:**
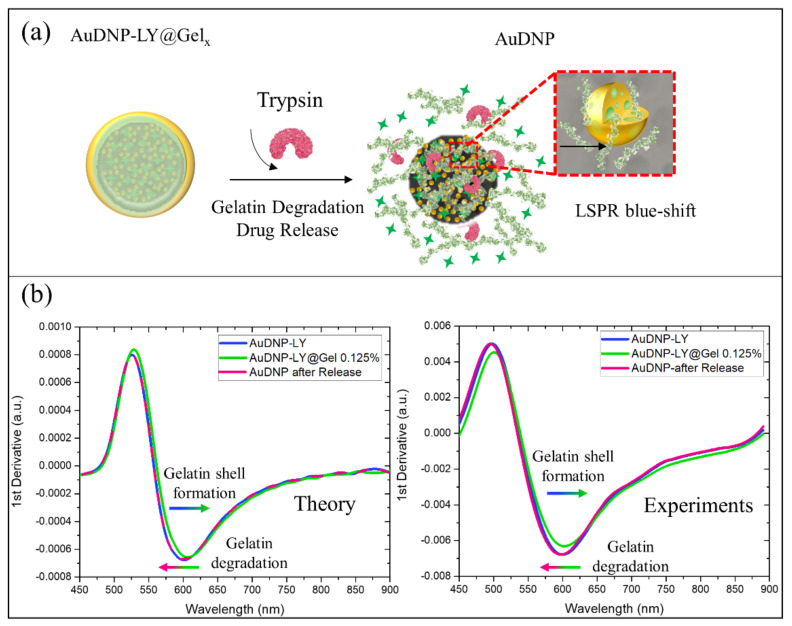
Gelatin degradation and drug release optical monitoring of AuDNP-LY@Gel_0.125%_. (**a**) Schematization of the in-vitro gelatin degradation and drug release in a solution containing the trypsin enzyme. The scheme is not in scale and is not intended to represent the full sample composition. (**b**) Theoretical and experimental first derivatives of the absorption spectra of the AuDNP-LY system (blue lines), AuDNP-LY@Gel_0.125%_ (green lines), and after drug release test, during which gelatin degradation occurs (purple lines).

As discussed in the previous sections, the complete optical modeling of the AuDNP-LY@Gel_x_ system could be used to monitor the gelatin shell formation on the surface of the nanocarrier in time. Furthermore, the presence of AuNPs on DNP provided our system with another important functionality: to monitor the degradation of the outer gelatin shell in presence of trypsin enzyme (Figure 5a) directly and to trace the drug release of LY indirectly. It was expected that once half of the gelatin thickness was degraded by trypsin, about half of the loaded drug in the nanocarrier was released; this could be evaluated by a spectroscopic measurement without the need for expensive equipment. As a proof of concept, the blue shift associated with the gelatin degradation of the AuDNP-LY@Gel_0.125%_ system was reported in Figure 5b.

From the theoretical point of view, trypsin enzymes used during the release studies reduced the medium effective refractive index from 1.54 (in presence of gelatin) to 1.33 (water refractive index) in the surroundings of AuNPs on the DNPs. For this reason, 100% of gelatin shell degradation would result in a blue shift in both $\;\lambda_{max\;}$ and $\lambda_{2\;}$ of the Gel-AuNPs absorption spectra. Ideally, the blue shift due to the gelatin degradation should be equal and opposite to the redshift caused by the gelatin shell formation on the AuDNP system. To show this, we reported in Figure 5b the theoretical first derivatives of the absorption spectra highlighting the perfect overlapping between blue and purple lines corresponding to the AuDNP system before the gelatin shell formation and after gelatin degradation, respectively.

To further validate our modeling for the gelatin degradation, we optically monitored the AuDNP-LY@Gel_0.125%_ system after the in-vitro release in presence of the trypsin enzyme. The experimental first derivatives of the absorption spectra were accurately predicted by our model (Figure 5b) since a blue shift of the inflection point in the first derivative occurred. However, from the experimental point of view, the overlap between the AuDNP-LY system (blue line) and AuDNP system after the drug release study (purple line), was not observed. The inflection wavelength of the AuDNP system underwent a slight blue shift compared to its initial position, after gelatin degradation. We attributed this phenomenon to the intrinsic hybrid nature of the Gel-AuNPs, which has been theoretically hypothesized and experimentally validated in this study. Indeed, as highlighted in the inset of Figure 5a, the AuNPs of this work contained gelatin inclusions, which could be partially available on their surface for trypsin recognition and degradation. Therefore, since the effective relative dielectric constant of Gel-AuNPs was strictly dependent on the gelatin volume fraction, also their optical properties could be affected by gelatin degradation. However, due to the very slight variation root mean square between theoretical and experimental results, this unwanted effect could be safely neglected.

## 3. Materials and Methods

### 3.1. Materials and Reagents

Diatomite was supplied by DEREF Spa (Castiglione in Taverina, Viterbo, Italy); 1-ethyl-3-[3-dimethylaminopropyl] carbodiimide-hydrochloride (EDC), N-hydroxy-succinimide (NHS), 3-aminopropyltriethoxysilane (APTES), type-B gelatin from bovine skin, tetrachloroauric acid (HAuCl_4_), sodium borohydride (NaBH_4_), sulfuric acid (H_2_SO_4_), trifluoroacetic acid (TFA), acetonitrile HPLC grade, Millex-GP syringe filter 0.22 μm and acetone were purchased from Sigma-Merck KGaA (Darmstadt, DE). Phosphate buffered saline (PBS) was purchased from GIBCO (Dublin, IRL). Hydrochloric acid (HCl) was purchased from Romil (Cambridge, UK). Absolute ethanol (EtOH) and hydrogen peroxide (H_2_O_2_) were purchased from Carlo Erba (Milano, IT). Galunisertib (LY 2157299) was purchased from Axon Medchem (Groningen, NL). All reagents were of analytical grade and all aqueous solutions were prepared with Milli-Q water.

### 3.2. Preparation of AuDNPs-LY@GEL System

As extensively described by our group in previous works, ultrasounds were applied to diatomite powder to reduce its average size to a nanometric scale, and a uniform distribution was obtained by repeating cycles of settling and recovery. Then, the DNPs were purified by HCl and Piranha solution to remove organic and inorganic contaminations [44]. The silanization of DNPs was carried out by immersing DNPs in 10% APTES ethanol solution for 30 min to allow the interaction between the silanol groups of DNPs and APTES. After silanization, DNPs were recovered by centrifugation (13,500 rpm, 15 °C, 10 min) and underwent a curing process for 1 h at 40 °C before collecting [21]. A dispersion of amino-modified DNPs (DNP-APT) was immersed in 1 mM HAuCl_4_ solution for 5 min and 0.5 mL of gelatin solution 0.025% was added as a AuNPs-stabilizer agent (weight ratio between gold salts and gelatin 10:1). The dispersion was stirred for further 5 min and an aqueous solution of 100 mM NaBH_4_ was added dropwise to the dispersion until the color turned from yellow to purple/red. Gold-decorated DNPs were left to rest for 30 min before purification by centrifugation (4500 rpm, 10 °C, 10 min). The supernatant was discarded and the gelatin stabilizing AuNPs was cross-linked by EDC and NHS in PBS solution (weight *ratio* gelatin: EDC: NHS = 10:14:7) for 90 min in mild stirring to weld AuNPs to the silica substrate firmly. At this stage, the cross-linked gelatin was considered as the surfactant stabilizing AuNPs and, therefore, we used low-concentrated gelatin solutions (0.025%). We will refer to the as-prepared NP dispersion as AuDNP. For drug loading, AuDNP dispersion was shacked in a $1\;\mathrm{mg}\cdot {\mathrm{mL}}^{-1}$ Galunisertib solution for 2 h at 37 °C and the excess of the unloaded drug was discarded after centrifugation at 4500 rpm. The dispersion was washed with water and PBS solution twice. We will refer to these NPs as AuDNPs-LY. The loaded system was then resuspended in gelatin aqueous solutions pH 4.0 at the following concentrations *w/v*: 0%, 0.125%, 0.25%, 0.5%, 0.75% and 1.50%. The mixture was stirred for 30 min, and then EDC and NHS were added for cross-linking the gelatin matrix (weight ratio gelatin: EDC: NHS = 10:14:7) for 1 h at room temperature. In this step, we used gelatin solutions more concentrated than the AuDNPs synthesis to promote the grow of a shell of the cross-linked matrix surrounding the silica substrate and protecting the system from drug dispersion phenomena. Finally, the dispersion was centrifuged, NPs were washed twice in water and collected for analysis (AuDNPs-LY@Gel_x_, where x is the different concentration in the gelatin shell).

### 3.3. Apparatus and Characterizations

All the characterizations were performed using 50 μg of DNPs in an aqueous solution in triplicate. Hydrodynamic diameter and surface ζ-potential of bare DNPs, AuDNPs, and AuDNPs-LY@Gel_x_ were measured using a Zetasizer Nano- ZS instrument (Malvern Instrument Ltd., Cambridge, UK) equipped with a He-Ne laser (633 nm, scattering angle of 90°, 25 °C). The morphology of DNPs after each surface modification was conducted using a FEI Tecnai 12 transmission electron microscope (FEI Company; Hillsboro, OR). Samples were prepared in water and incubated for 10 min on a standard copper grid (100 mesh) covered with a Formvar film. Next, the drop was removed, and the grid was air-dried overnight at room temperature. From TEM images, a particle analysis was performed by ImageJ software to estimate the Gel-AuNPs mean radius (*r*). Absorption spectra of amino-modified DNPs, AuDNPs-LY, and AuDNPs-LY@Gel_x_ were recorded on Cary 100 UV-Vis double beam spectrophotometer (Agilent, CA, USA) using quartz cells of 10 mm path at room temperature. Samples were dispersed in de-ionized water immediately before the measurement. The peak analysis and first derivatives of the measured spectra were performed by OriginPro 2016 (OriginLab, Northampton, MA, USA).

### 3.4. Numerical Simulations

We modeled each Gel-AuNP as a complex medium made of two constituents, namely gold and gelatin. Starting from the knowledge of their permittivities, and the measured absorption spectra, we identified the gelatin mean volume fraction $\mu_{Gel}\;$and the corresponding standard deviation $\sigma_{gel}$ through a genetic optimization [45,46]. A schematic of this identification is reported in Figure 2a. The fitness function to be minimized was the root mean square of the difference between the theoretical absorption spectrum ($C_{abs_{th}}$) and the mean of the experimental absorption spectrum ($C_{abs_{exp}}$) of Gel-AuNPs immobilized on DNPs. Thus, the objective function $F\left(\mu_{gel},\sigma_{gel}\right)$ of the minimization algorithm was the following:

(4) $$\left(\mu_{Gel},\sigma_{Gel}\right)=||C_{abs_{th}}\;\left(\lambda ,\;\varepsilon_{eff}\left(\mu_{Gel},\sigma_{Gel}\right)\right)-C_{abs_{exp}}\left(\lambda \right)||,$$

where λ is the operating wavelength, and $\mu_{Gel}$ and $\sigma_{Gel}$ are the mean and the standard deviation of the gelatin inclusions volume fractions ($f_{Gel}$) within the AuNPs. Gelatin inclusions were modeled as a gaussian distribution of spheres within the AuNPs. The GA returned the minimization of F as a function of the two variables $\mu_{Gel}$ and $\sigma_{Gel}$. The effective relative dielectric permittivity of Gel-AuNPs was obtained by the Maxwell-Garnett homogenization theory [35,36]. It consists of a weighted contribution of gold dielectric permittivity ($\varepsilon_{Au}$) measured by Johnson and Christie [47] and gelatin refractive index ($n_{Gel}=1.54)$:

(5) $$\varepsilon_{eff}\left(f_{Gel}\right)=\varepsilon_{Au}\left[1+\frac{3f_{Gel}\left(\frac{n_{Gel}^{2}-\varepsilon_{Au}}{n_{Gel}^{2}+2\varepsilon_{Au}}\right)}{1-f_{Gel}\frac{n_{Gel}^{2}-\varepsilon_{Au}}{n_{Gel}^{2}+2\varepsilon_{Au}}}\right]$$

In Equation (5),$\;f_{Gel}$ was obtained as a gaussian distribution with mean $\mu_{Gel}$ and standard deviation $\sigma_{Gel}$. The theoretical spectrum of Gel-AuNPs ($C_{abs_{th}}$) was predicted analytically by the Mie Theory [17,34]. The radius of AuNPs (r) was set to 3 nm, resulting from the particle analysis of TEM images, and the medium effective refractive index (n) in the AuNPs surroundings was assumed to be made of DNPs ($n_{Diatoms}=1.45$) and water ($n_{H_{2}O}=1.33$), in which the whole system (AuDNPs) was suspended during the spectroscopic measurements. The root mean square of the difference between $C_{abs_{th}}$ and $C_{abs_{exp}}$ was used as a fitness function and the optimization algorithm stopping condition was set at a value below 0.1, corresponding to 10% of deviation between theory and experiments. Then, a gelatin layer of thickness *t* was assumed to uniformly cover the Gel-AuNPs described so far. The absorption spectra of AuDNPs-LY@Gel systems with increasing gelatin-coating thicknesses *t* were obtained by the Mie-Kerker theory [48]. A redshift of the resonance peak was observed by increasing *t*. The first derivatives of the predicted spectra were computed and the theoretical optical response as a function of *t* and the experimental optical response as a function of gelatin concentration (%) were correlated. Validation of the proposed core-shell model was performed by predicting the experimental absorption spectra of core-shell Au@SiO_2_ NPs reported by Gontero et al. [49]. The numerical simulations were performed by Matlab (academic release, Mathworks, Natick, MA, USA, release R2020a). 

### 3.5. In Vitro Drug Loading and Release Studies

In vitro drug release of LY was performed in PBS solution containing the proteolytic enzyme trypsin to allow the digestion of the gelatin shell. For each sample with a different gelatin shell, 0.1 mg of AuDNP-LY@Gel_x_ were immersed in 1 mL of PBS solution containing 60 μg of trypsin (60 μg/mL) and stirred at 200 rpm at 37 °C. An excess of trypsin was used to guarantee that the same amount of gelatin was degraded in each sample at the same time intervals. After 5 min, the AuDNP-LY@Gel_x_ dispersion was centrifuged (3500 rpm, 37 °C, 5 min) to separate the NPs from the release solution. At each time interval, a freshly prepared PBS solution was added to the nanoparticle dispersion in presence of trypsin and stirred at 200 rpm, 37 °C until the following withdrawal. Release solutions were collected at time intervals of 5, 10, 20, 30, 60 min, and 16 h for the quantitative analysis by Reverse-Phase-High Performance Liquid Chromatography (RP-HPLC). Separation by liquid chromatography was obtained using a Shimadzu C18 Column as stationary phase (5 μm particle size, 250 × 4.6 mm) and a mobile phase composed of a water-acetonitrile (0.02% TFA) gradient in 20 min. The quantification of drug release was obtained by reading the sample absorbance peak at 254 nm and 9.2 min of retention time. For this quantitative analysis, a calibration curve of LY was previously performed. The cumulative release was calculated by using the following equation:

(6) $$Cumulative\;Release=R\left(t_{0}-1\right)+Rt_{0}$$

where *Rt*_0_ is the amount of the drug at time *t*_0_ and *R*(*t*_0_ − 1) is the amount of released drug at the previous time to t. The loaded drug was estimated as the total drug released after 16 h in a solution containing a large excess of trypsin (60 μg∙mL^−1^).

## 4. Conclusions

The recent trends in nanobiotechnology offer diverse strategies to raise the loading capacity of NPs employed in therapy. Among all these approaches, NPs can be coated by a polymeric shell that acts as a drug gatekeeper and enhances the carrier loading capacity. The present work shows that the drug loading capacity of diatomite-based carriers can be improved and tuned as a function of the gelatin shells around the biosilica surface. The Galunisertib loading capacity of the proposed nanocarrier AuDNP-LY@Gel is modulated from $2.4\;to\;5.6\;\mu g\cdot {\mathrm{mg}}^{-1}$ of NPs in response to the amount of gelatin in the outer shell. Our approach introduces the remarkable functionality to modulate the loading capacity of DNPs according to the therapeutic dose of the drug to encapsulate and deliver in the cell. The increment of the loading capacity enables lowering the administered mass of NPs in vitro, improving cell safety as well. Since the overexpression of gelatin-degrading enzymes is altered in the tumor microenvironment, the choice of gelatin as a capping material offers two advantages: it makes the nanocarrier loading capacity adjustable to the needs and the drug release triggerable by external *stimuli*. Moreover, the presence of AuNPs in our system provides the nanocarrier with optical features suitable for developing the full-optical modeling and monitoring the formation and degradation of gelatin shells spectroscopically. The localized plasmon resonance generated by noble nanostructures allows monitoring nanoparticle functionalization, overcoming the limitations of costly and standard methods. Herein, the formation of the gelatin shell on the surface of the AuDNP-LY@Gel system is confirmed by optical investigations and the shell thickness is estimated through numerical simulations. Furthermore, the gelatin degradation and subsequent drug release are confirmed from both theoretical and experimental points of view. Through this approach, we show that thicker gelatin shells provide DNPs with better loading capacity and retention functionalities, allowing the development of high-performing delivery systems. The opportunity to monitor gelatin degradation with an optically modeled system may promote the gelatin shells degradation and drug release monitoring directly in-vivo. Indeed, the LSPR response of differently shaped gold NPs could be tuned to stay in the invisibility region of human tissues, which falls in the range of 700–1100 nm. In this way, the optical response of plasmonic NPs could be followed spectroscopically without the need for expensive and laborious equipment. Similarly, the gelatin degradation profile—directly correlated to drug loading capacity—can be optically monitored to evaluate drug release within the targeted tissues.

## Data Availability

The data that support the findings of this study are available from the corresponding author upon reasonable request.

## Supplementary Materials

The following are available online at https://www.mdpi.com/article/10.3390/ijms221910755/s1, Figure S1—Morphological analysis of diatomite powder and Gel-AuNPs composition by TEM investigation; Figure S2—Validation of the homogenization modeling compared to experimental data; Figure S3—Optical monitoring of the drug loading step on the AuDNP system; Figure S4—Intrinsic absorptions of the single components of the AuDNP-LY@Gel system; Figure S5—pH-responsive behavior of the AuDNP-LY@Gel system in different buffer solutions; Figure S6—Experimental absorption spectra of AuDNPs with increasing gelatin concentrations; Figure S7—Validation of the core-shell model compared to standard Au@SiO_2_ NPs reported in the literature.
