# Supplementary material for: Design of Gelatin-Capped Plasmonic-Diatomite Nanoparticles with Enhanced Galunisertib Loading Capacity for Drug Delivery Applications
Source: Int J Mol Sci. 2021 Oct 5;22(19):10755. doi: 10.3390/ijms221910755 (PMC8509241; doi:10.3390/ijms221910755)
Supplement: Supplementary file 1 [file ijms-22-10755-s001.zip › ijms-1386549-supplementary.pdf]

Design of gelatin-capped plasmonic-diatomite nanoparticles with enhanced Galunisertib loading capacity for drug delivery applications.

Chiara Tramontano [1,2,†], Bruno Miranda [1,3,†], Giovanna Chianese [1], Luca De Stefano [1,*], Carlo Forestiere [3], Marinella Pirozzi [4] and Ilaria Rea [1]

[1] Institute of Applied Sciences and Intelligent Systems—Unit of Naples, National Research Council, Via Pietro Castellino 111, 80131 Naples, Italy; chiara.tramontano@na.isasi.cnr.it (C.T.); bruno.miranda@na.isasi.cnr.it (B.M.); giovanna.chianese@na.isasi.cnr.it (G.C.); ilaria.rea@na.isasi.cnr.it (I.R.)

[2] Department of Pharmacy, Università degli Studi di Napoli Federico II, Via Domenico Montesano 49, 80131 Naples, Italy

[3] Department of Electrical Engineering and Information Technology, Università degli Studi di Napoli Federico II, Via Claudio 21, 80125 Naples, Italy; carlo.forestiere@unina.it (C.F.)

[4] IEOS (Istituto per l'Endocrinologia e l'Oncologia Sperimentale) "G. Salvatore" Seconda Unità – CNR, Via Pietro Castellino, 111, 80131 Naples, Italy; m.pirozzi@ieos.cnr.it (M.P.);

* Correspondence: luca.destefano@cnr.it (L.D.S.)

† Chiara Tramontano and Bruno Miranda contributed equally to this work.

Keywords: diatomite; gold nanoparticles; gelatin; drug delivery; plasmonics; Galunisertib.

SUPPORTING INFORMATION

S1 – Morphological analysis of diatomite powder and Gel-AuNPs composition by TEM investigation

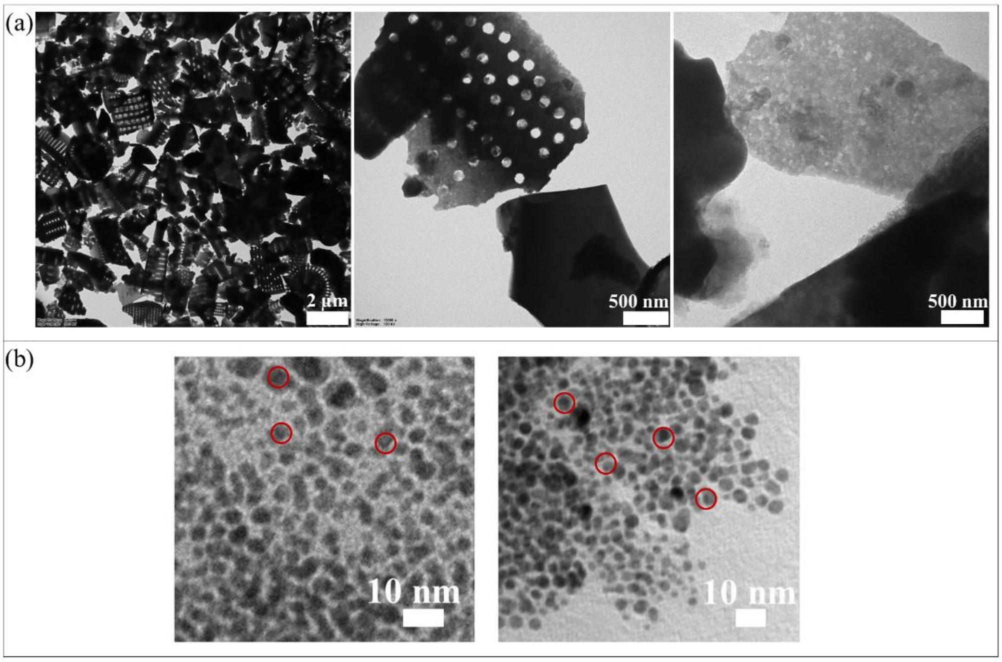

Figure S1: TEM images of diatomite powder (a) and Gel-AuNPs (b).

Diatomite powder is a mixture of different species of diatoms each one having a peculiar pore pattern that can be visible by TEM or not, as shown in Figure S1a. This variable diatomite composition is responsible for the heterogeneity of the DNP dispersion, which can be constituted by fragments with diverse porosity structures and sizes. In Figure S1b, the heterogeneity of Gel-AuNPs is shown. We attributed this non-homogeneous substructure of AuNPs to the presence of gelatin inhomogeneities, assumed as spherical inclusions in our optical modeling. Indeed, in the synthesis process, the nucleation of the AuNPs on DNP happens in presence of gelatin, which exhibits a higher refractive index than gold in the visible. Since the addition of sodium borohydride happens in a second moment, it is reasonable to assume that some molecules of gelatin could remain entrapped within the nanoparticles. Being the AuNPs much smaller than the operating wavelength, the approximation of the gelatin inclusions as spherical does not affect the prediction and evolution of the system optical response significantly. Therefore, even if the gelatin is not inside the particle, but on top of it, our model can still describe its experimental optical response with high accuracy.

S2 – Validation of the homogenization modeling compared to experimental data.

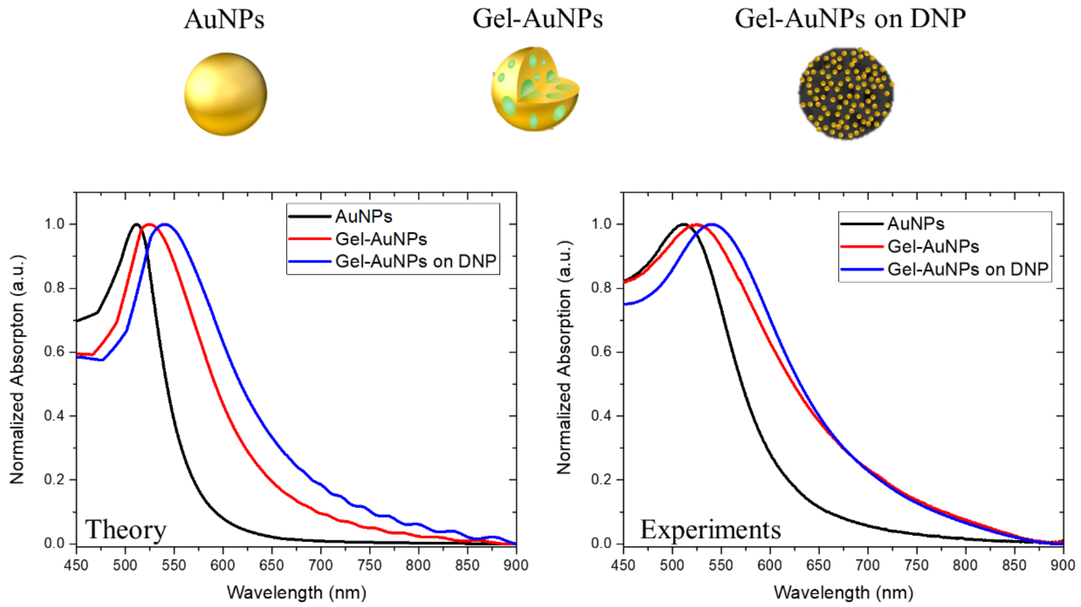

Figure S2: Normalized theoretical (left) and experimental (right) absorption spectra of 3 nm radius AuNPs, Gel-AuNPs, and Gel-AuNPs on DNPs.

The validation of the homogenization approach herein proposed, was performed by simulation of a 3 nm radius AuNP with no gelatin inclusions in water, a 3 nm radius Gel-AuNP in water, and a 3 nm radius Gel-AuNP on a silica substrate. The absorption of the three different systems is reported in Figure S2 (on the left). AuNP exhibited a LSPR peak at 512 nm, Gel-AuNP at 525 nm, and Gel-AuNP on silica at 540 nm. Accordingly, we experimentally evaluated the absorption of 3 nm radius AuNPs, synthesized by reducing gold ions by sodium borohydride, Gel-AuNPs obtained in presence of gelatin, and Gel-AuNPs on DNP obtained in presence of gelatin and diatomite in the reaction. The size of the first two samples was checked by DLS (confirming a distribution of AuNPs and Gel-AuNPs of mean radius 3 nm), while the distribution of Gel-AuNPs on DNP was obtained by TEM analysis, as shown in Figure 1c. The very good agreement between theory and experiments confirms the validity of our optical modeling.

S3 – Optical Monitoring of the Drug Loading step on the AuDNP system

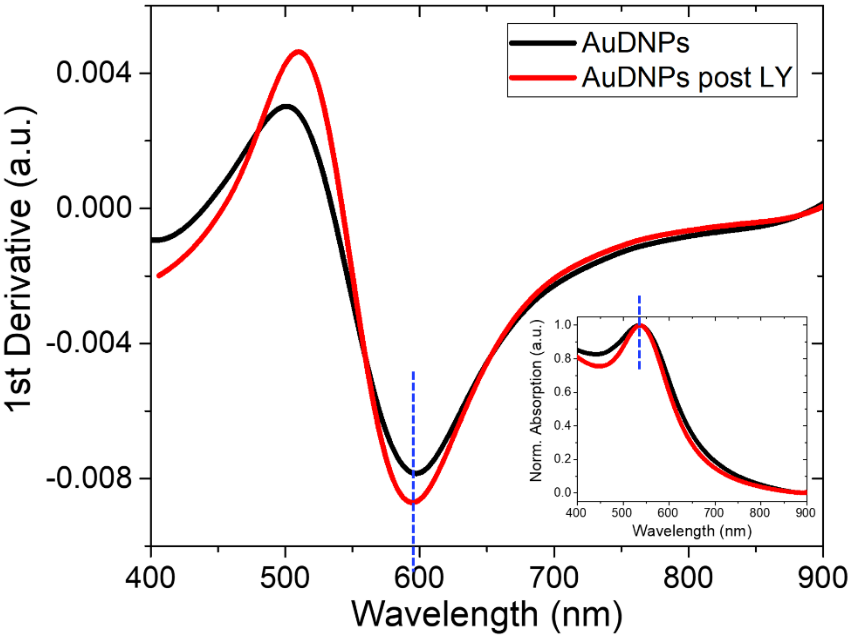

Figure S3: Optical monitoring of the drug loading step on the AuDNP system**:** Experimental first derivative and absorption spectra (in the inset) of AuDNP system before (black line) and after (red line) drug loading process.

Although the small molecule LY has a refractive index of 1.75, it did not cause any significant shift in the LSPR optical response of the AuDNP system neither in λ_{max} nor λ_2, due to the low molecular weight of the drug. Therefore, the loading step of LY was considered negligible for the modeling of our system.

S4 - Intrinsic absorptions of the single components of the AuDNP-LY@Gel system

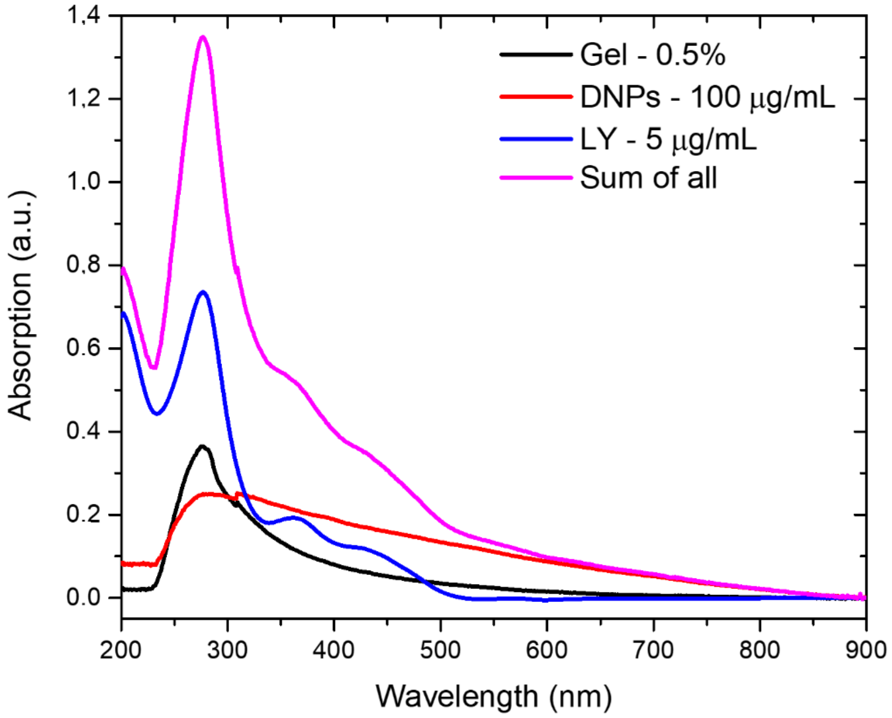

Figure S4: Intrinsic absorptions of the single components of the AuDNP-LY@Gel$_{0.5\%}$ system: absorption of Gelatin at a concentration 0.5 % (black line), DNPs at a concentration of 100 µg/mL (red line), and LY drug at a concentration of 5 µg/mL (blue line). The summation of all the contributes is also reported (purple line).

Figure S4 shows the UV-Vis absorbance measurements of a gelatin solution (0.5%), DNPs dispersion (100 µg/mL), and LY solution (5 µg/mL). The reported absorbances do not affect the optical response of the AuDNP-LY@Gel system in the resonant λ_{max} and the second inflection point λ_2 spectral positions. On the contrary, a tiny contribution is observed at the first inflection point λ_1, which causes a slight discrepancy (< 10%) between theoretical model and experimental measurements in that region.

S5 – The pH-responsive behavior of the AuDNP-LY@Gel

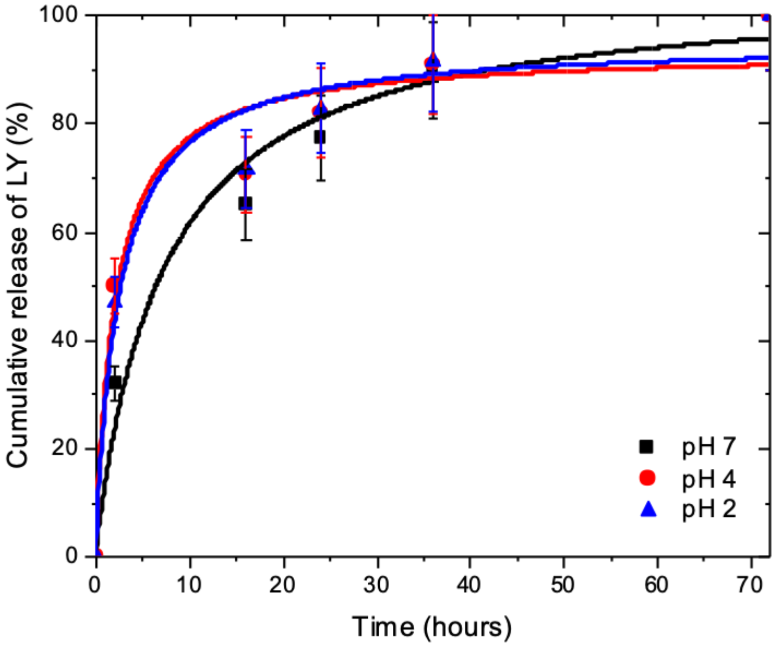

Figure S5: The pH-responsive behavior of the AuDNP-LY@Gel $_{0.25\%}$ system in solution in different buffer solutions: Drug release profile of AuDNP-LY@Gel 0.25% in Phosphate Buffered Saline (PBS) solution at pH 2.0 (blue line), 4.0 (red line) and 7.0 (black line) monitored for 70 hours.

The cumulative release of LY from AuDNP-LY@Gel 0.25% was quantified by Reversed-Phase High-Performance Liquid Chromatography (RP-HPLC). At neutral pH (7.0), the gelatin shell is tightly folded, and the drug release is delayed by the polymer matrix. In contrast, due to the sufficient extension of gelatin chains and the degradation of some gelatin molecules in the acidic environment (pH < 5), the release of LY is promoted in more acidic solutions. Indeed, 50% of the drug was released within 2.6 hours in acidic conditions (pH < 5), whereas the release of LY approached 50% of the maximum loaded drug within 6.5 hours in a neutral environment (pH = 7.0).

S6 – Experimental absorption spectra of AuDNPs with increasing gelatin concentrations

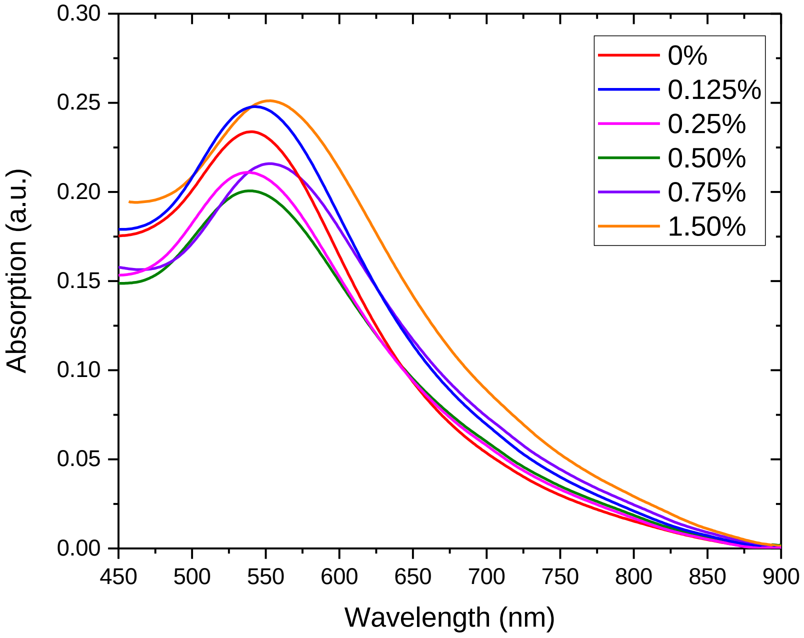

Figure S6: Experimental absorption spectra of AuDNPs with increasing gelatin concentrations before normalization: 0% (red), 0.125% (blue), 0.25% (purple), 0.5% (green), 0.75 % (violet), 1.50% (orange).

S7 – Validation of the core-shell model compared to standard Au@SiO_2 NPs reported in the literature

To further validate our core-shell modeling compared to what is already available in the literature, we considered the work by Gontero *et al.*,[1] in which citrate-AuNPs of diameter 41 nm with increasing shells of silica (SiO_2) were fabricated and optically characterized. A very good agreement between the reported experimental spectra and our modeling (Figure S7) was found in terms of accurate prediction of the LSPR position. This result suggests that our model could be easily adapted to different applications and heterogeneous systems.

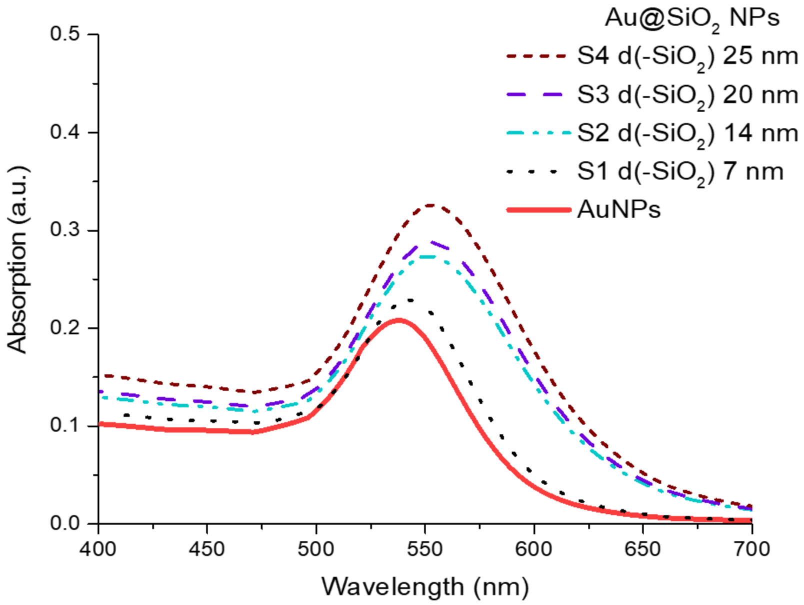

Figure S7: Theoretical absorption spectra of AuNPs and Au@SiO_2 core-shell NPs with increasing SiO_2 shell thicknesses: 20.5 nm -radius AuNPs (red), AuNPs with 7-nm thick SiO_2 shell (blue dots), AuNPs with 14-nm thick SiO_2 shell (light blue dash-dots), AuNPs with 20-nm thick SiO_2 shell (violet dashes), and AuNPs with 25-nm thick SiO_2 shell (magenta, short dashes).

References

1. Gontero, D.; Veglia, A. V.; Bracamonte, A.G.; Boudreau, D. Synthesis of ultraluminescent gold core-shell nanoparticles as nanoimaging platforms for biosensing applications based on metal-enhanced fluorescence. *RSC Adv.* **2017**, *7*, 10252–10258, doi:10.1039/c6ra27649k.
